# Factors Contributing to the Biofilm-Deficient Phenotype of *Staphylococcus aureus sarA* Mutants

**DOI:** 10.1371/journal.pone.0003361

**Published:** 2008-10-10

**Authors:** Laura H. Tsang, James E. Cassat, Lindsey N. Shaw, Karen E. Beenken, Mark S. Smeltzer

**Affiliations:** 1 Department of Microbiology and Immunology, University of Arkansas for Medical Sciences, Little Rock, Arkansas, United States of America; 2 Department of Biology, University of South Florida, Tampa, Florida, United States of America; Theodor-Boveri-Institut fur Biowissenschaften, Wurzburg, Germany

## Abstract

Mutation of *sarA* in *Staphylococcus aureus* results in a reduced capacity to form a biofilm, but the mechanistic basis for this remains unknown. Previous transcriptional profiling experiments identified a number of genes that are differentially expressed both in a biofilm and in a *sarA* mutant. This included genes involved in acid tolerance and the production of nucleolytic and proteolytic exoenzymes. Based on this we generated mutations in *alsSD*, *nuc* and *sspA* in the *S. aureus* clinical isolate UAMS-1 and its isogenic *sarA* mutant and assessed the impact on biofilm formation. Because expression of *alsSD* was increased in a biofilm but decreased in a *sarA* mutant, we also generated a plasmid construct that allowed expression of *alsSD* in a *sarA* mutant. Mutation of *alsSD* limited biofilm formation, but not to the degree observed with the corresponding *sarA* mutant, and restoration of *alsSD* expression did not restore the ability to form a biofilm. In contrast, concomitant mutation of *sarA* and *nuc* significantly enhanced biofilm formation by comparison to the *sarA* mutant. Although mutation of *sspA* had no significant impact on the ability of a *sarA* mutant to form a biofilm, a combination of protease inhibitors (E-64, 1-10-phenanthroline, and dichloroisocoumarin) that was shown to inhibit the production of multiple extracellular proteases without inhibiting growth was also shown to enhance the ability of a *sarA* mutant to form a biofilm. This effect was evident only when all three inhibitors were used concurrently. This suggests that the reduced capacity of a *sarA* mutant to form a biofilm involves extracellular proteases of all three classes (serine, cysteine and metalloproteases). Inclusion of protease inhibitors also enhanced biofilm formation in a *sarA/nuc* mutant, with the combined effect of mutating *nuc* and adding protease inhibitors resulting in a level of biofilm formation with the *sarA* mutant that approached that of the UAMS-1 parent strain. These results demonstrate that the inability of a *sarA* mutant to repress production of extracellular nuclease and multiple proteases have independent but cumulative effects that make a significant contribution to the biofilm-deficient phenotype of an *S. aureus sarA* mutant.

## Introduction

*Staphylococcus aureus* is an opportunistic pathogen capable of causing diverse forms of infection. Treatment of these infections is complicated not only by the continued emergence of antibiotic-resistant strains but also by the fact that many *S. aureus* infections are associated with formation of a biofilm, which limits the efficacy of antimicrobial therapy even in cases caused by strains that are not clinically defined as resistant to the relevant antibiotics [1], [2]. For this reason, the effective treatment of biofilm-associated staphylococcal infections often requires surgical debridement to remove infected tissues and/or devices [3], [4].

Previous reports have implicated many *S. aureus* genes in biofilm formation. These include *agr* [5], [6], *arlRS* [7], *bap* [8], *hla* [9], *ica* [10], *rbf* [11], *sarA* [12], [13], *sigB* [14], [15], *tcaR* [16], and *traP* [17], [18]. However, in almost all cases, there are conflicting reports with respect to the overall contribution of specific loci. For example, some reports have concluded that *ica* and *sigB* are required for *S. aureus* biofilm formation [10], [14], [15], while others have found that mutation of these loci has little impact [13], [19], [20]. Similarly, there is a report concluding that alpha hemolysin is required for biofilm formation [9], but *S. aureus* isolates unable to produce alpha toxin owing to a nonsense mutation in the corresponding gene (*hla*) are capable of forming a biofilm and causing biofilm-associated infection [19], [21]. In fact, a recent report concluded that non-hemolytic variants arise spontaneously within a biofilm and ultimately become the dominant subpopulation [22].

Such conflicting reports may be due to strain-dependent differences among *S. aureus* isolates. For instance, *bap* encodes a surface-associated protein (Bap) that promotes biofilm formation, but to date it has been found only in bovine mastitis isolates and even then only rarely [23], [24]. Many studies focusing on biofilm formation have also utilized strains derived from NCTC 8325. This includes RN6390, which is an 8325-4 strain in which three prophage were cured from NCTC 8325 [25], and SA113, which is a mutagenized, restriction-modification deficient derivative of 8325 [10], [26]. All 8325-derived strains carry natural mutations in *rsbU*, which renders them functionally *sigB* deficient [27]–[29]. They also carry a mutation in *tcaR* [16], a regulatory locus that has also been implicated in biofilm formation [30]. Perhaps owing to these mutations, the 8325-4 strain RN6390 has a reduced capacity to form a biofilm by comparison to clinical isolates of *S. aureus* [19], [21].

One exception to such conflicting reports is the staphylococcal accessory regulator (*sarA*), mutation of which has been consistently correlated with a reduced capacity to form a biofilm in both *S. aureus* and *S. epidermidis* [12], [13], [21]. Indeed, with the exceptions of RN6390 and Newman, the latter also being a poor biofilm former [12] that has specific characteristics (e.g. production of truncated fibronectin-binding proteins) that distinguish it from primary clinical isolates [31], mutation of *sarA* has resulted in a reduced capacity to form a biofilm *in vitro* in every *S. aureus* strain we have examined [12]. Mutation of *sarA* in the clinical isolate UAMS-1 was also shown to result in a significant decrease in biofilm formation *in vivo* as defined using a catheter-based murine model [19].

The *sarA* locus encodes a DNA-binding protein (SarA) that has a global impact on gene expression in *S. aureus* [21], [32], and it remains unclear which components of this global response are most relevant to biofilm formation. Mutation of *sarA* does result in reduced expression of the *ica* operon and consequently reduced production of the polysaccharide intercellular adhesion (PIA), but our direct comparison of *sarA* and *ica* mutants generated in the same strain used in the experiments reported here (UAMS-1) demonstrate that this cannot account for the biofilm defect in a *sarA* mutant [19].

As a first step toward defining the role of *sarA* in biofilm formation, we compared the regulons defined by growth within a biofilm and by mutation of *sarA*. We found that a large number of genes were differentially expressed in a mature biofilm by comparison to both exponential and post-exponential phase planktonic cultures [19] and that many of these were also in the *sarA* regulon [33]. Included among these genes was the bicistronic operon *alsSD*, which encodes the enzymes (acetolactate synthase and acetolactate decarboxylase) required for the conversion of pyruvate to acetoin rather than the more acidic products of glucose metabolism. Specifically, expression of *alsSD* was increased in a biofilm by comparison to both exponential and post-exponential planktonic growth [12]. Conversely, *alsSD* expression was decreased in a *sarA* mutant [33]. This suggests that the inability to express *alsSD* at adequate levels in a *sarA* mutant may contribute to its inability to form a biofilm.

Also included in both the biofilm and *sarA* regulons was *nuc*, which encodes the *S. aureus* thermostable nuclease. The expression pattern of *nuc* was opposite that of *alsSD* in that it was decreased in a biofilm but increased in a *sarA* mutant [19], [33]. This observation, together with recent reports demonstrating that extracellular DNA (eDNA) contributes to *S. aureus* biofilm formation [34], [35], suggest that the inability to repress production of extracellular nuclease may also contribute to the biofilm-deficient phenotype of a *sarA* mutant.

Expression of the genes encoding extracellular proteases was also altered both in a biofilm and a *sarA* mutant, but in this case the scenario is more complex in that expression of some protease genes (e.g. *scpA*) was decreased in a biofilm while expression of others (e.g. *sspAB*) was increased [19]. However, expression of all of these genes, as well as the gene encoding aureolysin (*aur*) was increased in a *sarA* mutant [33]. This is consistent with the observation that the overall production of extracellular proteases is increased in a *sarA* mutant [36]–[38]. This is potentially important in that biofilm formation in clinical isolates of *S. aureus* is facilitated by coating the substrate with plasma proteins [12], and the increased protease production observed in *sarA* mutants has been correlated with a reduced capacity to bind host proteins including fibronectin [36], [39]. Moreover, a recent report concluded that the *agr*-mediated induction of protease production limits biofilm formation and may play a functional role with respect to the dispersal of *S. aureus* from an established biofilm [40]. Such results suggest that the reduced capacity of a *sarA* mutant to form a biofilm could also be related to the increased production of one or more extracellular proteases.

Based on these considerations, we investigated the role of *alsSD*, *nuc*, and extracellular proteases in *S. aureus* biofilm formation with a specific emphasis on whether any or all of these factors contribute to the biofilm-deficient phenotype of a *sarA* mutant.

## Results And Discussion

### Impact of *sarA* on *alsSD* expression, acetoin production, stationary phase survival and biofilm formation

The *alsS* and *alsD* genes constitute a bicistronic operon and encode acetolactate synthase and acetolactate decarboxylase respectively. These enzymes function sequentially to convert pyruvate to 2-acetolactate and then acetoin, the latter ultimately being converted by acetoin reductase to 2,3- butanediol. Production of these neutral products rather than the more acidic products of carbohydrate metabolism is important for acid tolerance in a number of bacterial species [41]. This is consistent with the observation that *alsSD* is expressed at elevated levels in response to mild acid treatment of *S. aureus* [42]. Our previous demonstration that expression of *alsSD* is also increased in a biofilm by comparison to planktonic cultures is therefore consistent with the hypothesis that a central theme behind the adaptation of *S. aureus* to persistence within a biofilm is survival within the acidic environment associated with carbohydrate metabolism, particularly under low oxygen conditions [19]. It is also consistent with a recent report demonstrating that *Streptococcus mutans* is more acid tolerant when grown in a biofilm than when grown in planktonic culture [43]. Moreover, production of the enzymes required for acetoin production in *Vibrio cholerae* is co-regulated with expression of genes directly involved in the switch between motility and biofilm formation [41]. This co-regulation provides further support for the hypothesis that acetoin production and acid tolerance are important for biofilm formation in diverse bacterial species.

**Figure 1:**
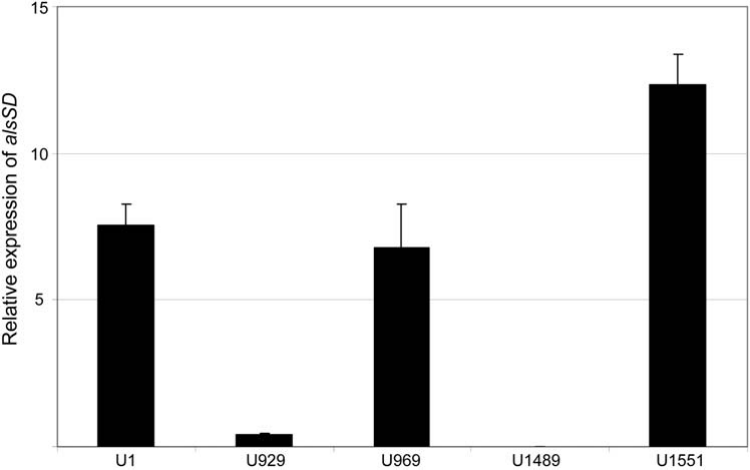
Expression of *alsSD* in *alsSD* and *sarA* mutants. The relative level of *alsSD* expression in each strain was determined by qRT-PCR. Results represent the mean±standard deviation of 3 replicate samples. Strain designations are: U1, UAMS-1 (parent strain); U929, UAMS-1 *sarA* mutant; U969, complemented *sarA* mutant; U1489, UAMS-1 *alsSD* mutant, U1551, complemented *alsSD* mutant.

**Figure 2 pone-0003361-g002:**
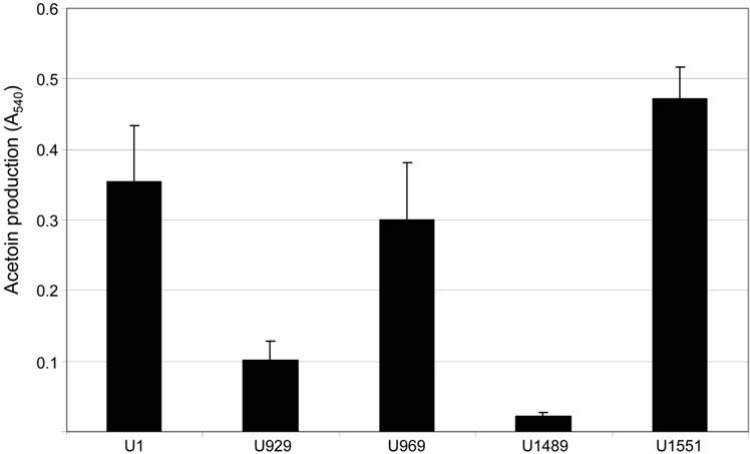
Production of acetoin and 2,3-butanediols in *alsSD* and *sarA* mutants. 18-hour culture supernatants were assayed for acetoin production using the Voges-Proskauer assay. Strain designations are the same as those cited in Fig. 1 and detailed in Table 1. Results represent the mean±standard deviation of 6 replicate experiments.

**Figure 3 pone-0003361-g003:**
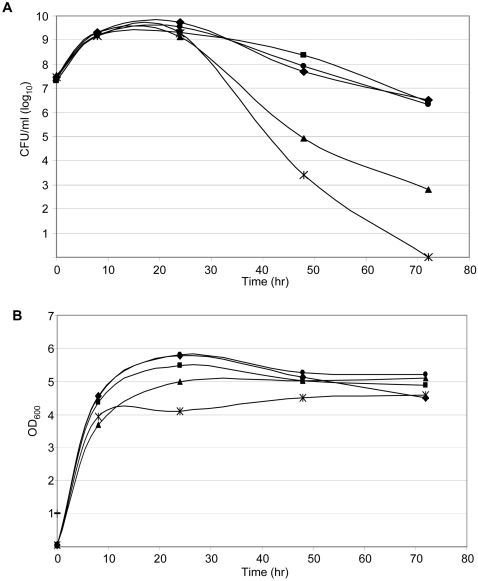
Effect of *alsSD* and *sarA* mutations on stationary phase survival. Each strain was grown in the presence of 35 mM glucose. Aliquots were removed at the indicated times to assess the number of colony-forming units (CFU) per ml (panel A) and culture density (panel B). Strain designations: UAMS-1 (▪), UAMS-929 (▴), UAMS-969 (•), UAMS-1489 (X), UAMS-1551 (♦).

**Figure 4:**
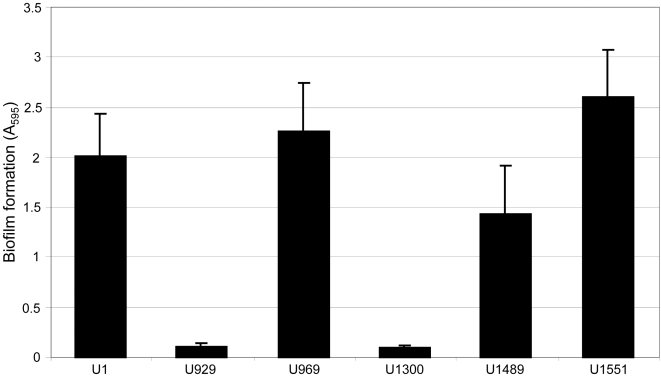
Effect of *alsSD* and *sarA* mutations on biofilm formation. Biofilm formation was assessed using the microtiter plate assay. Results represent the mean and standard deviation of 24 replicates. Strain designations are the same as those in Figs. 1–3 with the addition of the *sarA/alsSD* double mutant (UAMS-1300).

A previous report described construction of a UAMS-1 *alsSD* mutant (KB1097) and concluded that mutation of *alsSD* eliminated acetoin production and resulted in reduced murein hydrolase activity and reduced stationary phase survival [44]. We subsequently demonstrated that KB1097 also has a reduced capacity to form a biofilm that is comparable to that observed with a UAMS-1 *sarA* mutant [33]. However, it was later discovered that KB1097 was a contaminant that ultimately proved to be *S. hominis* rather than *S. aureus* (Dr. Ken Bayles, personal communication). Based on this, we independently generated an *alsSD* mutant (UAMS-1489) using the pKOR1 mutagenesis system [45]. Characterization of this mutant confirmed the absence of *alsSD* transcription (Fig. 1), the inability to produce acetoin and 2,3-butanediol as defined by the Voges-Proskauer assay (Fig. 2), and reduced stationary-phase survival (Fig. 3). The results also confirmed that mutation of *sarA* impacts all three phenotypes in a similar although less definitive manner (Figs. 1–3). Additionally, the capacity to form a biofilm was significantly reduced in UAMS-1489 by comparison to the UAMS-1 parent strain (p<0.001) (Fig. 4). Transcription of *alsSD*, the Voges-Proskauer phenotype, stationary-phase survival, and biofilm formation were all complemented by introducing a functional *alsSD* operon into UAMS-1489 to generate UAMS-1551 (Figs. 1–4). These results are consistent with the hypothesis that *alsSD* plays a role in biofilm formation in *S. aureus*.

While UAMS-1489 had a reduced capacity to form a biofilm, it remained significantly greater than that observed with the UAMS-929 *sarA* mutant (p<0.001) (Fig. 4). This was true despite the fact that mutation of *alsSD* had a greater impact than mutation of *sarA* on *alsSD* transcription, the Voges-Proskauer assay, and stationary-phase survival (Fig. 1–4). This demonstrates that the impact of *sarA* on biofilm formation extends beyond its impact on transcription of *alsSD*. Confirmation of this was obtained by analysis of the *sarA/alsSD* double mutant (UAMS-1300), which demonstrated that concomitant mutation of *alsSD* and *sarA* had no further impact on the production of acetoin (data not shown) but did reduce biofilm formation even by comparison to an *alsSD* mutant (*p*<0.001) (Fig. 4).

**Figure 5:**
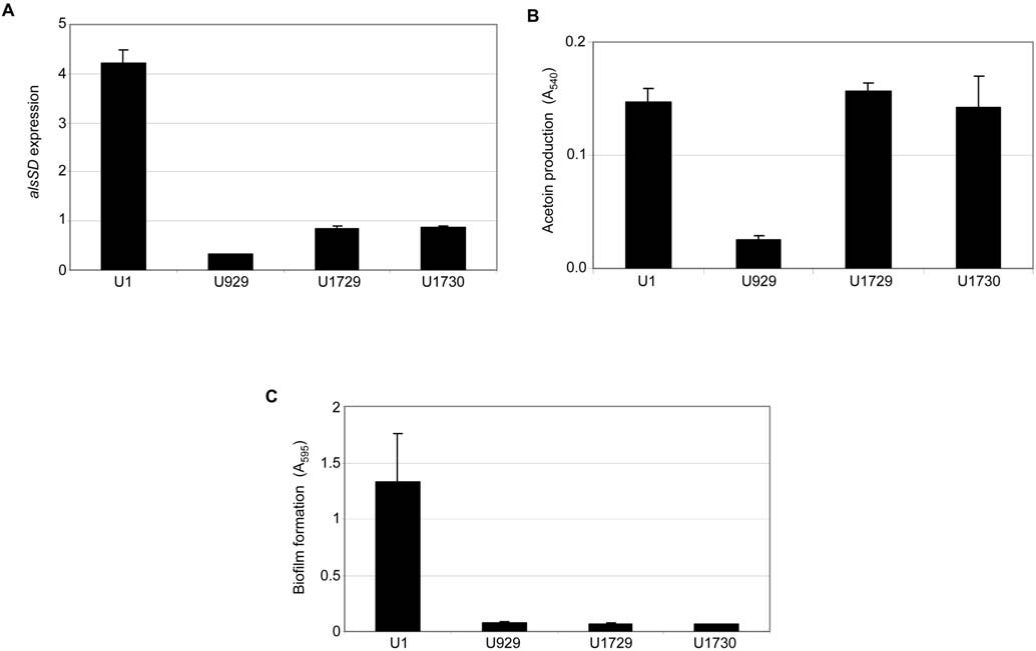
Impact of *alsSD* expression in a *sarA* mutant. Panel A: Expression of *alsSD* was assessed by qRT-PCR. Results represent the mean±standard deviation of the results obtained with 3 replicate samples. Panel B: 18-hour culture supernatants were assayed for acetoin production using the Voges-Proskauer assay. Results represent the mean±standard deviation of 6 replicate experiments. Panel C: Relative levels of biofilm formation were determined using the microtiter plate assay. Results represent the mean±standard deviation of 16 replicate experiments. Strain designations are the same as in previous figures with the addition of UAMS-1729 (U1729) and UAMS-1730 (U1730), both of which are UAMS-1 *sarA* mutants complemented with pLI50::*alsSD*.

Although these results demonstrate that the impact of *sarA* on *alsSD* transcription cannot fully account for the biofilm-deficient phenotype of a *sarA* mutant, mutation of *alsSD* did have a significant impact on biofilm formation by comparison to the parent strain (Fig. 4), and this leaves open the possibility that the impact of *sarA* on *alsSD* transcription makes an important contribution in that regard. Based on this, we also generated derivatives of UAMS-929 (UAMS-1729 and UAMS-1730) in which *alsSD* expression was enhanced in a *sarA* mutant. Although this did not fully restore *alsSD* transcription to wild-type levels (Fig. 5A), which might be expected given the positive impact of *sarA* on *alsSD* transcription [33], it did restore transcription to levels sufficient to fully restore the Voges-Proskauer phenotype (Fig. 5B). However, it had no impact on the ability of a *sarA* mutant to form a biofilm (Fig. 5C). This provides further support for the conclusion that mutation of *sarA* results in a defect in biofilm formation that extends beyond its impact on expression of *alsSD*.

One possible explanation for these results is that *alsSD* is part of a pathway that includes acetoin reductase, which is required to convert acetoin to 2,3-butanediol, and expression of the corresponding gene (SA0239 in the N315 genome) was also reduced in a *sarA* mutant [33]. Thus, restoration of acetoin production in a *sarA* mutant may not fully restore the ability to convert pyruvate to 2,3-butanediol. While such an effect might not be evident in the Voges-Proskauer assay, it could nevertheless compromise other aspects of metabolism including the ability to maintain pH homeostasis. However, subsequent analysis of all relevant strains failed to reveal any significant difference in the pH of culture supernatants from overnight cultures grown in biofilm medium, which includes exogenous glucose (data not shown). The alternative explanation is that *sarA* has an impact on biofilm formation that involves genes unrelated to the acetoin/2,3-butanediol pathway.

### Contribution of thermostable nuclease to the biofilm-deficient phenotype of a *sarA* mutant

Previous genome-scale transcriptional profiling experiments focusing on UAMS-1 identified a number of other *sarA*-regulated genes that were also differentially expressed in a biofilm by comparison to planktonic cultures [19], [33]. Included among these genes was *nuc*, which encodes the *S. aureus* thermostable nuclease. Specifically, expression of *nuc* was reduced in a biofilm and increased in a *sarA* mutant [12], [33]. Recent reports demonstrating that extracellular DNA contributes to biofilm formation in *S. aureus* [34], [35] are consistent with the hypothesis that this may also contribute to the biofilm-deficient phenotype of a *sarA* mutant.

**Figure 6:**
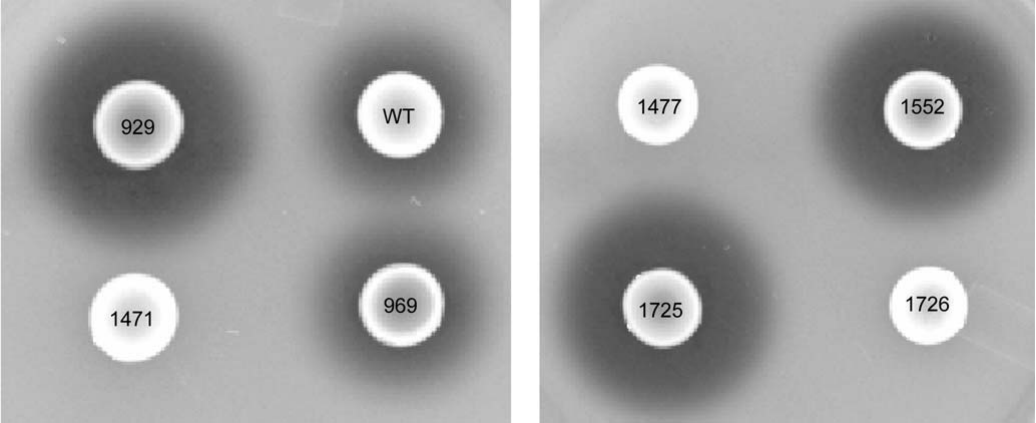
Production of extracellular nuclease. Results were assessed after overnight incubation of the indicated strains overnight on DNase test agar plates. Numbers refer to UAMS strain designations (Table 1).

**Figure 7:**
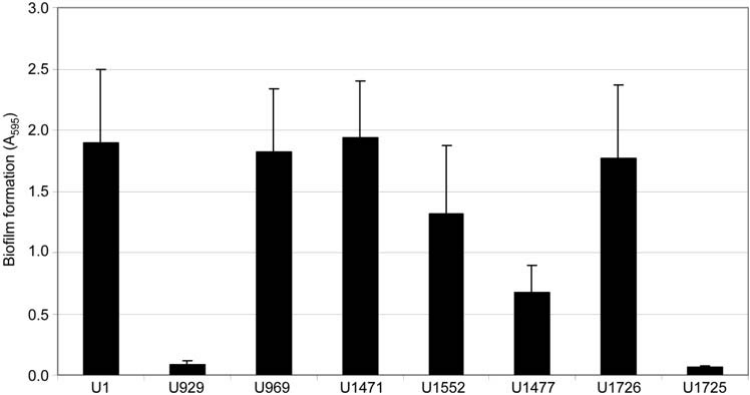
Effect of nuclease on biofilm formation. Biofilm formation was assessed using the microtiter plate assay. Results represent the mean±standard deviation of 24 replicates. Strains designations are the same as in previous figures with the addition of UAMS-1725 and UAMS-1726, which are the *sarA/nuc* mutant complemented with *nuc* and *sarA* respectively.

To address this hypothesis, we also generated UAMS-1 *nuc* and *sarA/nuc* mutants and evaluated the impact on biofilm formation. Phenotypic assays confirmed that mutation of *sarA* increased nuclease production and that it was completely abolished by mutation of *nuc* even in a *sarA* mutant (Fig. 6). Mutation of *nuc* in UAMS-1 (UAMS-1471) had no impact on biofilm formation, but complementation of the *nuc* mutation with a plasmid-borne version of the *nuc* gene (UAMS-1552) did limit biofilm formation to a significant degree (*p* = 0.001) (Fig. 7), presumably because it resulted in increased production of nuclease by comparison to the parent strain (Fig. 6). More importantly, concomitant mutation of *nuc* in a *sarA* mutant (UAMS-1477) enhanced biofilm formation by comparison to the *sarA* mutant (*p*<0.001) (Fig. 7). This effect was completely reversed by complementation of the *sarA/nuc* double mutant with a plasmid-borne version of the *nuc* gene (UAMS-1725) (Fig. 7). At the same time, biofilm formation in the *sarA/nuc* mutant (UAMS-1477) was not restored to wild-type levels. In contrast, complementation of the *sarA/nuc* mutant with *sarA* did fully restore biofilm formation (Fig. 7). These results indicate that increased production of extracellular nuclease contributes to the biofilm-deficient phenotype of a UAMS-1 *sarA* mutant but that additional factors must also be involved.

### Contribution of extracellular proteases to the biofilm-deficient phenotype of a *sarA* mutant

Also included in both the biofilm and *sarA* regulons were most of the *S. aureus* genes encoding extracellular proteases, the only exception being the *spl* operon (*splABCDEF*), expression of which was largely unaltered both in a biofilm and in a *sarA* mutant. For example, expression of *scpA* was decreased in a biofilm but elevated in a *sarA* mutant. Expression of the gene encoding aureolysin (*aur*) was not altered in a biofilm but was increased in a *sarA* mutant [19], [33]. Expression of the *sspABC* operon was elevated in a biofilm and a *sarA* mutant, but because no direct comparison has been made, it is not known whether expression of *sspABC* is elevated in a *sarA* mutant even by comparison to a biofilm.

It is clear that overall extracellular protease activity is elevated in *sarA* mutants [36]–[38], but to date studies that have attempted to address the issue have concluded that this has relatively little impact on the biofilm-deficient phenotype of a *sarA* mutant. For instance, mutation of *sspA*, which encodes the classic “V8” serine protease (SspA), had no impact on biofilm formation in either UAMS-1 or its *sarA* mutant [33]. Similarly, Valle *et al.* (2003) concluded that the increased production of proteases in *sarA* mutants was unlikely to explain their biofilm-deficient phenotype based on the observations that 1) the capacity of a *sarA* mutant to form a biofilm was not enhanced in the presence of the protease inhibitors α_2_-macroglobulin or E64, 2) the capacity of the wild-type strains (ISP479C and 15981) to form a biofilm was not reduced by incubation in concentrated supernatants from *sarA* mutants, and 3) concomitant mutation of *sarA* and *sspA* or *sarA* and the aureolysin gene (*aur*) failed to enhance biofilm formation.

In contrast to such studies, Boles and Horswill (2008) recently concluded that elevated protease production can be correlated with reduced biofilm formation and that the increased production of extracellular proteases plays an important role in *agr*-mediated dispersal from an established biofilm. These studies were limited to derivatives of SH1000, which is an *rsbU*-repaired derivative of 8325-4, and they did not address the potential contribution of extracellular proteases to the biofilm-deficient phenotype of *sarA* mutants, but such results nevertheless suggest that previous studies may not have fully defined the impact of protease production in this context. Based on this, we employed a combination of three protease inhibitors in an attempt to collectively limit the activity of all recognized *S. aureus* extracellular proteases. Specifically, E-64, 1-10-phenanthroline, and dichloroisocoumarin (DIC) inhibit the activity of cysteine proteases (e.g. ScpA and SspB), metalloproteases (e.g. aureolysin), and serine proteases (e.g. SspA and the Spl proteases) respectively.

**Figure 8:**
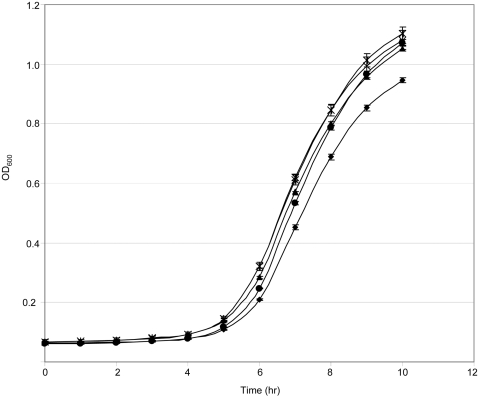
Effect of protease inhibitors on growth. Growth of the UAMS-929 *sarA* mutant in biofilm medium without protease inhibitors (♦) was compared to growth in the same medium containing 1 mM DIC (▴), 1 mM E-64 (•), 10 µM 1,10-phenanthroline (+) or a cocktail containing all three inhibitors (X). Growth was monitored at hourly intervals for 10 hours. Results indicate the OD_600_±standard deviation of 3 replicates.

**Figure 9:**
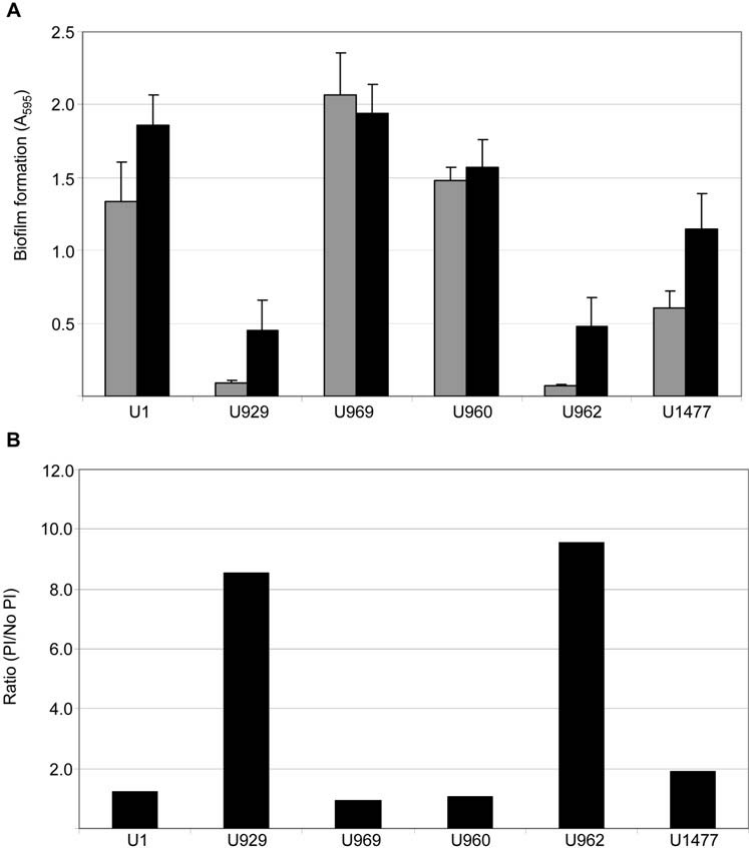
Effect of protease inhibitors on biofilm formation. Panel A: The microtiter plate assay of *in vitro* biofilm formation was performed with bacteria grown in biofilm medium (gray) or biofilm medium containing the inhibitor cocktail (black). Results represent the mean±standard deviation of 24 replicates. Panel B: Results of the biofilm assay shown in panel A represented as the ratio of biofilm formation in the presence of the inhibitor cocktail/biofilm formation in the absence of inhibitors.

Each inhibitor was added to the medium used in our biofilm assays at a concentration of 1 mM or, in the case of phenanthroline, the highest concentration (10 µM) that did not limit growth (Fig. 8). In fact, addition of these inhibitors both alone or in combination with each other increased growth of the *sarA* mutant to a limited degree at least as defined by the maximum optical density of post-exponential phase cultures (Fig. 8). This suggests that the increased production of proteases may actually limit the growth of an *S. aureus sarA* mutant. Subsequent analysis using skim milk-agar plates confirmed that this combination of protease inhibitors also decreased overall proteolytic activity by comparison to the *sarA* mutant (data not shown). More importantly, at least in the context of this report, inclusion of these inhibitors also enhanced biofilm formation in a *sarA* mutant (*p*<0.001) (Fig. 9A). Although a slight increase in biofilm formation was also observed with the parent strain, analysis of the relative effects confirmed that the impact in a *sarA* mutant significantly exceeded that observed in UAMS-1 (Fig. 9B). These results indicate that the increased production of extracellular proteases also makes an important contribution to the biofilm-deficient phenotype of a UAMS-1 *sarA* mutant.

**Figure 10:**
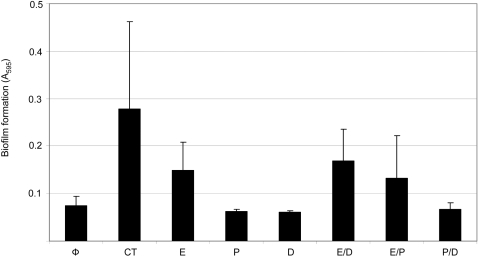
Biofilm formation in the presence of individual inhibitors alone and in combination with each other. Biofilm formation in the UAMS-929 *sarA* mutant was assessed in the absence of protease inhibitors (Φ), the presence of the protease inhibitor cocktail (CT), or in presence of individual inhibitors alone and in paired combinations with each other. Inhibitor designations are E-64 (E), DIC (D) and phenanthroline (P). Results represent the mean±standard deviation of 24 replicates.

One possible explanation is that the presence of protease inhibitors increased growth of the *sarA* mutant, particularly with respect to the maximum density observed with post-exponential phase cultures (Fig. 8). However, we observed a similar degree of growth enhancement when each inhibitor was included alone as well as in combination with each other, and this was not the case with respect to biofilm formation. Specifically, E-64 was the only individual inhibitor in which a significant increase in biofilm formation was observed either alone or in combination with DIC or phenanthroline (*p*<0.001) (Fig 10). This suggests that the effect of the inhibitor cocktail on biofilm formation cannot be explained by its impact on growth of the *sarA* mutant in biofilm medium. It also indicates that the cysteine proteases ScpA and/or SspB play an important role. This is in contrast to the results of Valle *et al.* (2003), who concluded that E-64 has no impact on the biofilm-deficient phenotype of a *sarA* mutant. However, the concentration of E-64 employed in these earlier experiments (10 µM) was considerably lower than that used in our experiments (1 mM). At the same time, the impact of E-64 even at the higher concentration was limited by comparison to both the wild-type strain (data not shown) and the *sarA* mutant grown in the presence of all three inhibitors (*p* = 0.012) (Fig. 10), and this clearly suggests that *sarA*-regulated proteases other than ScpA or SspB are also involved.

In an effort to further define the role of specific proteases, we also examined the impact of mutating *sspA* in both UAMS-1 (UAMS-960) and its *sarA* mutant (UAMS-962). In both cases, mutation of *sspA* had no significant impact on biofilm formation (Fig. 9A). Additionally, the enhanced biofilm formation observed with a UAMS-1 *sarA* mutant grown in the presence of all three inhibitors was also observed with a *sarA/sspA* double mutant (*p*<0.001) (Fig. 9B). This indicates that the impact of the protease inhibitors on biofilm formation was independent of SspA. This is consistent with the results of Valle *et al.* (2003), who also found that mutation of *sspA* had no impact on biofilm formation in a *sarA* mutant.

**Figure 11:**
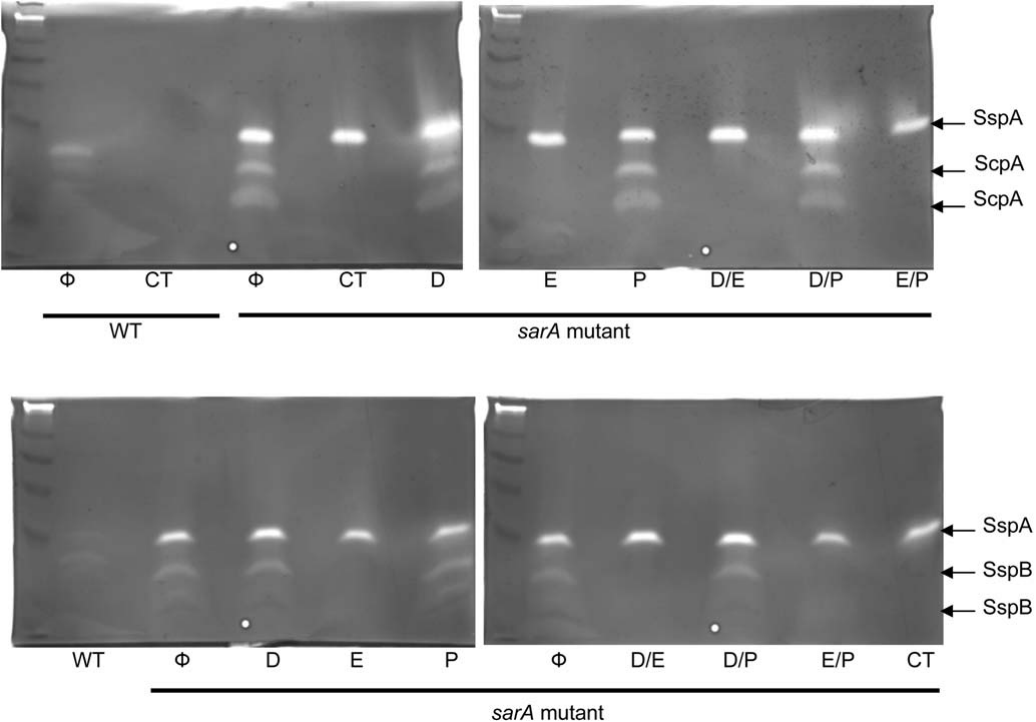
Zymogram analysis of protease production in the presence of protease inhibitors. Analysis of the production of specific proteases was assessed using casein (upper panel) and gelatin gels (lower panel) in UAMS-1 (WT) and its UAMS-929 *sarA* mutant. Analysis was done after growth in biofilm medium in the absence of any inhibitor (Φ), the presence of the inhibitor cocktail (CT), or the presence of each inhibitor alone and in combination with each other. Inhibitor designations are the same as in the legend for Fig. 10. The presumed identity of individual proteases is indicated to the right. In the case of SspA, this was confirmed by analysis of the corresponding *sarA/sspA* mutant (data not shown). In the case of ScpA and SspB, identification is presumptive based on their identity as cysteine proteases, inhibition of both by E-64, and the fact that ScpA has greater activity than SspB on casein while the opposite is true on gelatin (Dr. Jan Potempa, personal communication).

To further examine this issue, we carried out zymogram analysis using both casein and gelatin gels. This analysis confirmed that both the inhibitor cocktail and E-64 alone or in combination with either DIC or phenanthroline reduced the activity of cysteine proteases to undetectable levels as defined by the sensitivity of our zymogram assays (Fig. 11). In contrast, we could not demonstrate significant inhibition of serine or metalloproteases with DIC or phenanthroline. Moreover, production of SspA was unaffected by the inclusion of DIC in the growth medium (Fig. 11). To the extent that DIC is a specific inhibitor of serine proteases, including SspA, this suggests that the concentration of DIC used in our experiments was below the level required to have a phenotypic effect. We could not determine whether the same was true for the *spl*-encoded serine proteases because we could not detect the activity of these enzymes in either of our zymograms. This is perhaps not surprising since the activity of these proteases was recently shown to be dependent on their binding of specific substrates in a manner analogous to the exfoliative toxins [46], [47].

Similarly, we could not demonstrate inhibition of the metalloprotease aureolysin with phenanthroline because we could not detect the activity of this protease even in a *sarA* mutant (Fig. 11). Nevertheless, the fact that the combination of all three proteases inhibitors enhanced biofilm formation to an extent that exceeded that observed with any single or even dual combination of protease inhibitors (Fig. 10) suggests that the concentrations of both DIC and phenanthroline used in these experiments were in fact inhibitory. It is also consistent with the hypothesis that extracellular proteases other than the cysteine proteases ScpA and SspB also make an important contribution to the biofilm-deficient phenotype of a *sarA* mutant. To the extent that this effect was independent of *sspA* (see above), this suggests that the *spl*-encoded serine proteases and/or aureolysin may be particularly important in this regard. Although confirmation of this hypothesis will require examination of UAMS-1 *sarA/aur* and *sarA/spl* mutants, this is consistent with the results of Boles and Horswill (2008), who detected increased levels of serine proteases in the effluent from *S. aureus* biofilms and demonstrated that concomitant mutation of *aur* and *spl* resulted in an enhanced capacity to form a biofilm at least in the 8325-4 strain SH1000.

While the combination of protease inhibitors enhanced biofilm formation in a *sarA* mutant, it did not restore it to wild-type levels (Fig. 9A). One explanation for this is that the concentration of inhibitors used in our experiments was sufficient to limit protease activity in a *sarA* mutant but not sufficient to restore it to wild-type levels. As discussed above, this is particularly true with respect to DIC and phenanthroline. It was not possible to use either of these inhibitors at a higher concentration either due to issues related to limited solubility in biofilm medium (DIC) or inhibition of growth (phenanthroline), so addressing this possibility will also require detailed analysis of relevant protease mutants. Nevertheless, the results discussed above suggest that the increased production of both extracellular nuclease and multiple extracellular proteases contribute to the biofilm-deficient phenotype of an *S. aureus sarA* mutant. However, they also demonstrate that the biofilm-defect in a *sarA* mutant cannot be explained by its impact on either nuclease or proteases alone. To determine whether these effects might be cumulative, we also examined whether inclusion of protease inhibitors had an impact on bioflm formation with a *sarA/nuc* mutant. The results confirmed that the effect was cumulative in that biofilm formation was enhanced in a *sarA/nuc* mutant in the presence of protease inhibitors by comparison to both a *sarA* mutant in the presence of protease inhibitors (*p*<0.001) and a *sarA/nuc* mutant in the absence of inhibitors (*p*<0.001) (Fig. 9A). In fact, in the presence of the inhibitor cocktail, the *sarA/nuc* mutant exhibited a level of biofilm formation approaching that of the UAMS-1 parent strain.

### Conclusions

Taken together, our results provide important clues with respect to explaining the biofilm-deficient phenotype of a *S. aureus sarA* mutant, and there may in fact be a common theme that ties them all together. For instance, a recent report demonstrated that mutation of *cidA*, which encodes a regulator of murein hydrolase activity, results in reduced release of extracellular DNA and a reduced capacity to form a biofilm [35]. The *cidA* gene is part of the *cidR* regulon, which also includes *alsSD* [44]. Our results confirmed that mutation of *alsSD* in UAMS-1 results in a stationary-phase survival defect manifested as cell death in the absence of cell lysis. This suggests that mutation of *alsSD* may limit the release of extracellular DNA as well as limit the capacity to produce acetoin and maintain pH homeostasis. In fact, one explanation for our results is that mutation of *alsSD* limits biofilm formation because it limits the availability of extracellular DNA but that this effect is modest by comparison to the impact of *sarA* on the production of extracellular nuclease. Such a scenario might also provide an alternative explanation for why restoration of *alsSD* expression in a *sarA* mutant failed to restore biofilm formation since what little extracellular DNA would be available would presumably be degraded due to the increased production of nuclease.

How the production of proteases might fit into this scenario remains undefined. The production of active extracellular nuclease does require protease processing [48], but if this were a defining characteristic of the biofilm-deficient phenotype of a *sarA* mutant, then mutation of *nuc* and inclusion of protease inhibitors would not be expected to have a cumulative effect since mutation of *nuc* eliminates the production of nuclease in any form. This suggests that nuclease and protease have independent effects that remain to be defined but are perhaps related to the attachment vs. accumulation phases of biofilm formation. Given our experimental approach using a cocktail of protease inhibitors, it also remains to be determined exactly what proteases are involved, how they interact with each other, what *S. aureus* proteins are the most relevant targets, and what role these targets play in biofilm formation. In this respect it should be noted that biofilm formation in UAMS-1 and other clinical isolates is enhanced by coating the substrate with plasma proteins [12] and that, at least in UAMS-1, it is not dependent on PIA production [19]. Moreover, we have demonstrated that the increased production of proteases in *sarA* mutants results in a decreased capacity to bind host proteins including fibronectin [36]. Taken together, these results suggest that the negative impact of proteases on biofilm formation may be multifactorial and involve both attachment and accumulation. A recent report also demonstrated that induction of *agr* expression leading to increased production of multiple proteases may also serve as a specific means of dispersal from an established biofilm [40].

While our experimental focus is on the role of *sarA* in biofilm formation, our results may also have broader implications for *S. aureus* regulatory circuits. For instance, *sarA* represses production of many *S. aureus* exoproteins including nuclease and multiple proteases [33], [36] while the accessory gene regulator (*agr*) has the opposite effect [25], [40]. While there are reports concluding that reduced expression of *agr* can be correlated with a reduced capacity to form a biofilm [18], [49]–[51], most studies have concluded that *agr* expression limits rather than enhances biofilm formation [5], [12], [52]. In fact, it has been proposed that induction of *agr* expression may serve as a specific means of dispersal from an established biofilm [5], [12], [22], [40].

One report suggested that the negative impact of *agr* on biofilm formation may be due to the increased production of delta-toxin, which is a phenol-soluble modulin (PSM) proposed to act as a surfactant to limit cellular accumulation [5], [53]. The alternative explanation is that expression of *agr* results in the increased production of both extracellular nuclease and protease(s) [25]. This would presumably promote detachment and release from a mature biofilm based on degradation of the relevant *S. aureus* adhesins and/or extracellular matrix components including extracellular DNA. Indeed, Boles and Horswill (2008) recently concluded that *agr*-mediated protease production plays a primary role in this regard. Together with our results, this would suggest that *sarA* and *agr* have independent and opposite effects on biofilm formation, with the ultimate impact being dependent on the relative contribution of each with respect to the other.

In this scenario, *S. aureus* strains that express *agr* at high levels would presumably have a reduced capacity to form a biofilm. This is consistent with our observation that the 8325-4 strain RN6390, which by comparison to most clinical isolates including UAMS-1 expresses *agr* at high levels and produces elevated amounts of nuclease and extracellular proteases [36], has a limited capacity to form a biofilm [12]. It is also consistent with the observation that mutation of *agr* enhances biofilm formation in RN6390 [12]. This effect is reversed by concomitant mutation of *sarA* [12], which suggests that the impact of *sarA* is epistatic to *agr* at least in this context.

It is unclear whether this has any relevance with isolates other than RN6390, which as noted above has specific characteristics that distinguish it from clinical isolates of *S. aureus*. However, a recent report concluded that most community-acquired MRSA (CA-MRSA) isolates express *agr* at higher levels than their healthcare-associated MRSA (HA-MRSA) counterparts [53]. Our studies done with a USA300 CA-MRSA isolate suggest that this does not preclude biofilm formation at least in this strain [52], but there are reports demonstrating a general inverse relationship between the level of *agr* expression and biofilm formation [5]. Together with the fact that *agr* expression is also correlated with the increased production of *S. aureus* extracellular proteins and PSMs, the latter having both surfactant-like and anti-phagocytic properties [53], this could perhaps explain why CA-MRSA isolates often cause acute infections while HA-MRSA tend to cause chronic infections that are more likely to have a biofilm-associated component [54].

Finally, our previous studies characterizing the *sarA* and biofilm regulons in UAMS-1 [19], [33] identified 43 genes that were differentially expressed both in a biofilm and in a *sarA* mutant [12], [33]. Of the four possible scenarios, 17 genes were expressed at lower levels in both a biofilm and a *sarA* mutant, 6 genes were expressed at higher levels in both a biofilm and a *sarA* mutant, 17 genes were expressed at lower levels in a biofilm and higher levels in a *sarA* mutant, and 3 genes were expressed at higher levels in a biofilm and lower levels in a *sarA* mutant. Given the complex and interactive nature of regulatory circuits in *S. aureus*, it is difficult to predict which of these scenarios would be most important, and we certainly do not preclude the need to investigate additional genes in the *sarA*/biofilm regulon. However, our results suggest that the inability of a *sarA* mutant to repress production of specific extracellular proteins, including nuclease and multiple proteases, play a particularly important role in that regard. A detailed understanding of these processes is important given the role of biofilms not only in the development of many forms of *S. aureus* infection but also with respect to their impact on the ability to effectively treat these infections.

## Materials And Methods

### Bacterial strains and growth conditions

The strains utilized in this study are listed in Table 1. All strains were maintained as stock cultures at −80°C in tryptic soy broth (TSB) containing 25% (v/v) glycerol. For each experiment, the relevant strains were retrieved from cold storage by plating on tryptic soy agar (TSA) with appropriate antibiotic selection. Antibiotics were used at the following concentrations: kanamycin (Kan; 50 µg per ml), neomycin (Neo; 50 µg per ml), chloramphenicol (Cm; 10 µg per ml), and erythromycin (Erm 5 µg per ml). To ensure that the results of phenotypic assays were consistent, all assays other than nuclease production (see below) were done using TSB supplemented with 0.5% glucose and 3.0% sodium chloride (biofilm medium) without antibiotic selection as previously described [12].

**Table 1:** Strains used in this study.

Strain	Description	Reference
UAMS-1	MSSA, osteomyelitis isolate	Gillaspy *et al.*, 1995
UAMS-929	UAMS-1*sarA::kan*	Blevins *et al.*, 2002
UAMS-960	UAMS-1*ssp::tet*	Blevins et al., 2002
UAMS-962	UAMS-929*/ssp::tet*	Blevins et al., 2002
UAMS-969	UAMS-929(pLI50::*sarA*)	Blevins *et al.*, 2002
UAMS-1300	UAMS-929Δ*alsSD*	This study
UAMS-1471	UAMS-1Δ*nuc*	This study
UAMS-1477	UAMS-929Δ*nuc*	This study
UAMS-1489	UAMS-1Δ*alsSD*	This study
UAMS-1551	UAMS-1489 (pLI50::*alsSD*)	This study
UAMS-1552	UAMS-1471 (pLI50::*nuc*)	This study
UAMS-1725	UAMS-1477 (pLI50::*nuc*)	This study
UAMS-1726	UAMS-1477 (pLI50::*sarA*)	This study
UAMS-1729	UAMS-929 (pLI50::*alsSD*)	This study
UAMS-1730	UAMS-929 (pLI50::*alsSD*)	This study

### Mutagenesis

Mutagenesis of *alsSD* and *nuc* was done as previously described [52] using the pKOR1 mutagenesis system [45]. According to the annotation for the MRSA252 genome, which was previously shown to be the most closely related of the sequenced strains to UAMS-1 [21], the specific open-reading frames (ORFs) targeted in these experiments were SAR2297 (*alsS*), SAR2296 (*alsD*), and SAR0947 (*nuc*). The corresponding ORFs in the N315 genome are SA2008/SA2007 (*alsSD*) and SA0746 (*nuc*). The oligonucleotide primers used for mutagenesis are listed in Table 2. In UAMS-1489, the deleted region started 650 bp downstream of the *alsS* start codon and ended 182 bp downstream of the *alsD* stop codon (primers alsSDMut1FattB1, alsSDMut1RSacII, alsSD2FSacII, alsSD2RattB2).

**Table 2:** Primers used in this study.

Primer or Probe	Oligonucleotide Sequence
*alsSD* Mut1FattB1	GGG GAC AAG TTT GTA CAA AAA AGC AGG CTC ACA CCA ATC AAT CCA ACA TCC C
*alsSD* Mut1RSacII	ATC GTA GCC GCG GTC AGC ACT AGA ACT TCT CAT ACC
*alsSD* 2FSacII	ATC GAT CCG CGG ATA TGC AAC TGT AAC TAA ATT CG
*alsSD* 2RattB2	GGG GAC CAC TTT GTA CAA GAA AGC TGG GTA TAA ATA AAT CCC CTC ACT ACC G
*nuc* Mut1FattB1	GGG GAC AAG TTT GTA CAA AAA AGC AGG CTG TAA GTA CAC TTA GTC AGT CTC ACC
*nuc* Mut1RSacII	GGA CCT CCG CGG CGA AAC ATT ACT GAT AGC CAT CCC T
*nuc* Mut2FSacII	GGA CCT CCG CGG TGA TAA ATA TGG ACG TGG CTT AGC G
*nuc* Mut2RattB2	GGG GAC CAC TTT GTA CAA GAA AGC TGG GTG GCC TTC TTC TAA TGA TTT GTA TCC
*alsSD* proF	CAG TCA TTT ATA TTC ATT TCC CTT C
*alsSD* downstream KpnI	GGA CCT GGT ACC CTA TGA CAA CCA TGC TTA ACC G
*nuc* comp-F	ACT TTG CTA AAG CTA CTG CAA AGG
*nuc* comp-R	TAA CTC ACA TTT TTC TTC ACG CTC
*alsSD* probe	CAT CTG TTT CAT AGC CCT CTT TAA TTG CCG
*alsSD* RTA	AAG GTT TAC GAG TTA CTA ATC AAG
*alsSD* RTS	AAT TTA CAG GTA TAT CAA TTA ATA CTG G
*gyrB2* probe	CCG CCA CCG CCG AAT TTA CCA CCA
*gyrB* RTA	CCA ACA CCA TGT AAA CCA CCA GAT
*gyrB* RTS	AGT AAC GGA TAA CGG ACG TGG TA

In the case of nuclease, the deleted region starts 83 bp downstream of the *nuc* start codon and ends 180 bp upstream of the *nuc* stop codon (primers nucMut1FattB1, nucMut1RSacII, nucMut2FSacII, nucMut2RattB2). Genotypic confirmation of all mutations was obtained by PCR using primers flanking the deleted region (data not shown). Because pKOR1-generated mutations are not marked by an antibiotic-resistance gene and cannot be transduced, mutagenesis of *alsSD* and *nuc* was done in both UAMS-1 and its corresponding *sarA* mutant (UAMS-929). The UAMS-1 *sspA* (SAR1022/SA0901) and *sarA/sspA* mutants were generated by Φ11-mediated transduction of the *ssp::tet* mutation from an existing 8325-4 mutant (kindly provided by Simon Foster, University of Sheffield) into UAMS-1 and UAMS-929 respectively. Generation of UAMS-929 is described elsewhere (Blevins et al., 2002).

### Complementation of *alsSD, nuc* and *sarA* mutations

Complementation of the *alsSD* mutation was done by cloning the region spanning 431 bp upstream and 118 bp downstream of the *alsSD* operon (primers alsSDproF and alsSD downstream KpnI) into the *E. coli/S. aureus* shuttle vector pLI50 [55]. Complementation of the *nuc* mutation was done by cloning the region spanning 453 bp upstream of 277 bp downstream of the *nuc* gene (primers nuc comp-F and nuc comp-R) into pLI50. Complementation of the *sarA* mutation was done as previously described [36]. In all cases, complementing plasmids were first introduced into the *S. aureus* strain RN4220 before transduction into the corresponding UAMS-1 mutants using Φ11 as previously described [56].

### Phenotypic assays

The Voges-Proskauer assay was used to assess the production of acetoin and 2,3-butanediol as previously described [44]. Briefly, cultures were grown for 18 hrs in biofilm medium (TSB supplemented with 0.5% glucose and 3.0% NaCl) with constant shaking before harvesting cell-free supernatants by centrifugation. The assay was done in 96-well microtiter plates by adding 50 µl of 0.3% creatine and 60 µl of 5% alpha-naphthol freshly prepared in 100% ethanol to 120 µl of fresh culture supernatant. After gentle mixing, 30 µl of 40% KOH was added to the reaction mixture, which was then incubated at room temperature for 30–45 min with occasional mixing. Results were assessed by measuring absorbance at 540 nm.

Biofilm formation was assessed *in vitro* using the static, microtiter plate biofilm assay as previously described [12]. To assess stationary-phase survival, overnight cultures of each *S. aureus* strain were grown in TSB with appropriate antibiotic selection and then used to inoculate 21 ml of NZY broth (Fisher Scientific, St. Louis, MO) containing 35 mM glucose to an OD_600_ of 0.05. Flasks were loosely capped and grown at 37°C with constant shaking. After 8, 24, 48, and 72 hrs, the optical density (OD_600_) of each culture was determined and an aliquot was removed to determine viable count by plating on TSA.

Nucleolytic activity was assessed using D'NASE Test Agar (REMEL, Lenexa, KS). Briefly, overnight cultures were standardized to an equal optical density before spotting 10 µl aliquots onto nuclease test agar. Plates were incubated overnight at 37°C. Nuclease activity was then assessed by overlaying the agar with 1N HCl to precipitate undigested DNA and define the zone of clearance around each strain.

Overall protease activity was assessed using skim milk agar as previously described [40], the only difference being that we analyzed standardized culture supernatants after 15-fold concentration using Centricon YM-3 filter units (Millipore, Bedford, MA). The activity of specific proteases was assessed by zymography using Ready Gel Zymogram Gels containing gelatin or casein (BioRad Laboratories, Hercules, CA). For casein gels, supernatants were analyzed without further processing. For gelatin gels, supernatants were first concentrated as discussed above. Samples in both cases were loaded onto gels in a buffer containing DTT but without β-mercaptoethanol. After electrophoresis, gels were first incubated for 30 min at room temperature (RT) in renaturing buffer (2.5% TritonX-100) and then overnight at 37°C in developing buffer (0.2 M Tris, 5 mM CaCl_2_, 1 mM DTT). To visualize protease bands, gels were then stained with SimplyBlue SafeStain (Invitrogen, Carlsbad, CA) at RT for 2 hrs before destaining overnight with water.

### Protease inhibitors

In experiments employing E-64 (Fisher Scientific, St. Louis, MO), and dichloroisocoumarin (DIC) protease inhibitors (Sigma Chemical Co., St. Louis, MO), each inhibitor was dissolved in biofilm medium at a 1 mM concentration. For experiments employing 1-10-phenanthroline (Fisher Scientific, St. Louis, MO), the concentration was reduced to 10 µM because higher concentrations inhibited growth. Subsequent experiments confirmed that these concentrations did not inhibit growth either alone or in combination with each other.

### RNA isolation and qRT-PCR analysis

To assess relative levels of *alsSD* expression, total bacterial RNA was isolated using the Qiagen RNeasy Mini Kit as previously described [52]. Quantitative, real-time RT-PCR (qRT-PCR) was then performed [33] using *alsSD*-specific primers and a corresponding TaqMan probe (Table 2). Results were standardized by comparison to the results obtained with the same samples using primers and a TaqMan probe corresponding to the *gyrB* gene (Table 2).

### Statistical analysis

Statistical comparisons were done using the Student's t-test or, where appropriate, the Mann Whitney Rank Sum Test as formatted in SigmaStat Statistical Software Version 2 (SPSS Inc., Chicago, IL). Because multiple comparisons were made within each data set, statistical significance, along with the corresponding *p* value, is noted in the text rather than within each figure.
